# Momentum-Based Adversarial Attacks and Multi-Level Denoising Defenses in Deep Learning-Based Wind Power Forecasting

**DOI:** 10.3390/s26072073

**Published:** 2026-03-26

**Authors:** Yangming Min, Congmei Jiang, Kang Yang, Xiankui Wen, Kexin Chen

**Affiliations:** 1 College of Electrical Engineering, Guizhou University, Guiyang 550025, China; 2 Guizhou University Survey and Design Institute Co., Ltd., Guiyang 550025, China; 3 Electric Power Science Research Institute of Guizhou Power Grid Co., Ltd., Guiyang 550002, China

**Keywords:** adversarial attack, deep learning, multi-level iterative denoising autoencoder, momentum iterative fast gradient sign method, wind power forecasting

## Abstract

Deep learning (DL) techniques have significantly advanced wind power forecasting by enhancing accuracy. However, these DL models are vulnerable to adversarial attacks, which can lead to severely inaccurate forecasts. Existing studies in wind power forecasting have rarely addressed the stealthiness and effectiveness of adversarial attacks simultaneously, nor have they investigated defense strategies against multiple perturbation strengths or in black-box scenarios. To this end, we propose an attack algorithm for wind power forecasting, i.e., the momentum iterative fast gradient sign method (MI-FGSM). This algorithm generates adversarial samples by incorporating momentum into the iterative process and adding perturbations to the input samples along the gradient direction. To defend against such attacks under varying perturbation strengths, a defense model called multi-level iterative denoising autoencoder (MLI-DAE) is proposed. MLI-DAE is trained using adversarial samples with multiple perturbation levels to effectively restore attacked inputs to their clean forms. Experimental results under both white-box and black-box scenarios demonstrate that MI-FGSM induces significantly larger forecast errors with smaller perturbation magnitudes compared to FGSM. Furthermore, our proposed MLI-DAE effectively defends against multi-level perturbations without compromising the original forecast accuracy.

## 1. Introduction

Conventional power generation’s heavy reliance on fossil fuels leads to environmental pollution and resource depletion and exacerbates global warming [1]. To mitigate these detrimental impacts and facilitate the transition towards a sustainable power system, renewable power generation has emerged as a crucial alternative [2]. Among these, wind energy has emerged as a widely used, environmentally friendly renewable resource for large-scale power generation [3]. However, wind power generation is subject to various factors such as wind speed, wind direction, and temperature, resulting in considerable randomness and variability [4,5]. Therefore, accurate wind power forecasting is essential for power management and planning, effectively enhancing the economic and social benefits of power systems [6,7].

In recent years, driven by the ongoing advancement of artificial intelligence, a myriad of machine learning (ML) techniques has been extensively applied to wind power forecasting [8]. Traditional ML forecasting models, such as support vector machine regression (SVR) [9], random forest [10], and autoregressive integrated moving average (ARIMA) [11], have been widely employed. However, these conventional techniques tend to be relatively simple and heavily reliant on manually engineered features, which can limit their predictive accuracy when dealing with complex and nonlinear wind power data. Recently, deep learning (DL) has emerged as the mainstream of ML, owing to its exceptional capacity to handle nonlinear mappings [12,13,14]. This inherent ability to automatically extract correlations between input samples and wind power output often results in more accurate forecasts [15]. Deep learning-based models, such as Long Short-Term Memory Networks (LSTMs) [16], Convolutional Neural Networks (CNNs) [17], Recurrent Neural Networks (RNNs) [18], Bidirectional Long Short-Term Memory (Bi-LSTM) [19], and Gated Recurrent Units (GRUs) [20], have wide application in wind power forecasting.

Although DNN-based forecasting models have achieved high accuracy, their nonlinear nature also makes them vulnerable to adversarial attacks [21,22,23]. Input data such as wind speed, wind direction, and ambient temperature are critical to accurate forecasts and are typically obtained via online weather forecast application programming interfaces (APIs) [22]. During transmission over communication networks, such data may be intercepted or tampered with, exposing potential attack surfaces for adversarial manipulation [23]. If the input data used for wind power forecasting are compromised by such attacks, the resulting perturbations can lead to degradation in forecasting accuracy, which may have real-world consequences, including inaccurate dispatching, increased reserve requirements, and reduced economic benefits for wind farm operators. In Ref. [24], the fast gradient sign method (FGSM) is employed to attack wind speed and wind direction data. The experimental results demonstrate that FGSM outperforms the civil attack (CA) in reducing forecast accuracy. In Ref. [25], the projected gradient descent method is utilized to perform non-directional, semi-directional and fully directional attacks on wind power data, and the advantages of various attack methods are extensively studied. In Ref. [26], an attack strategy targeting external-factor data is proposed. This approach employs an attack sample selection model to improve stealthiness by selectively filtering the attack samples and an attack direction judgment model to enhance the attack effectiveness by determining the correct attack direction. In Ref. [27], a new attack algorithm, called the adversarial learning attack, is proposed for wind power forecasting. This algorithm stably optimizes the meteorological data into its adversarial patterns, effectively degrading the forecast accuracy. Although existing studies have demonstrated that wind power forecasting models are vulnerable to adversarial attacks, most research has not simultaneously addressed both the effectiveness and the stealthiness of attacks.

To ensure the safe application of DNNs in wind power forecasting, it is crucial to investigate the defense algorithms. The adversarial training (AT) [25,28] is an advanced defense algorithm that improves the robustness of DNNs by retraining them on a mixed set comprising both adversarial and clean samples. In Ref. [29], SSA is used to attack DNN to generate adversarial PQD signals, and then the DNN performs adversarial training through these signals. However, traditional adversarial training often performs poorly when dealing with a range of perturbation strengths and can even degrade the model’s original forecast accuracy. In Ref. [30], the iterative adversarial training (IAT) is proposed in PQD classification. This method employs multiple perturbation training defense models, significantly enhancing the model’s ability to defend against attacks of varying perturbation strengths. The DAE method performs preprocessing before the forecasting model, effectively mitigating the impact of attacks without significantly compromising the original forecast accuracy [31]. In Ref. [32], the DAE defense is employed for preprocessing, effectively safeguarding deep learning-based power allocation models from adversarial attacks in massive MIMO systems without compromising original forecast accuracy. However, DAE performs optimally only in defending against an attack with a single strength. In wind power forecasting, there is little research on defense algorithms. In Ref. [24], a preprocessing framework for wind power forecasting is proposed to defend against adversarial attacks in the white-box environment. This method mitigates attacks by identifying perturbations in input samples and replacing corrupted samples with corresponding forecasted values. In Ref. [25], the effectiveness of adversarial training is validated in the field of wind power forecasting, emphasizing its capacity to enhance model robustness in the white-box environment. Currently, research on adversarial defenses in wind power forecasting has yet to explore the effectiveness of attacks involving multiple perturbation strengths. Additionally, existing studies have not explored the defense issues in the black-box environments.

**Figure 1:**
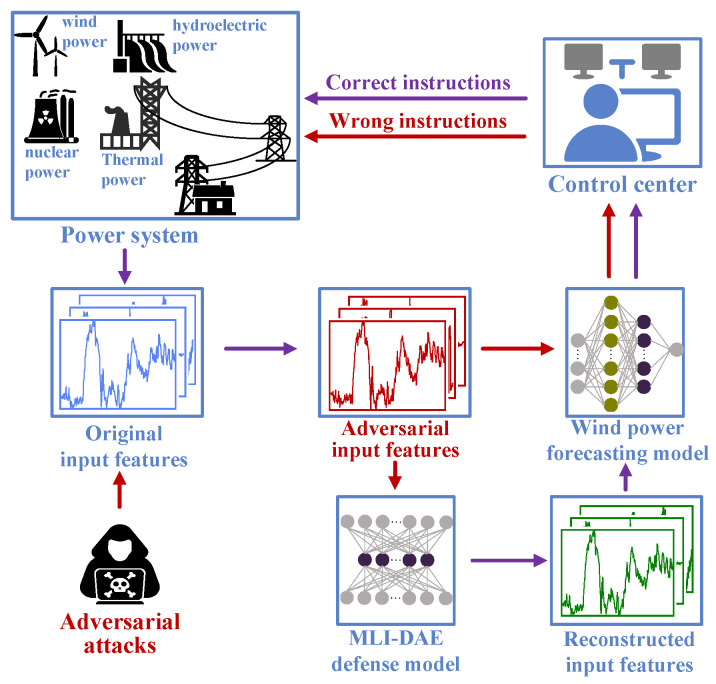
Adversarial attack and defense in wind power forecasting system.

Overall, research on adversarial attacks and defenses in wind power forecasting remains limited, and the security aspects of this field warrant further in-depth investigation. Although momentum-based attacks, such as the momentum iterative fast gradient sign method (MI-FGSM), have been extensively studied in other domains, their application to wind power forecasting, along with the associated challenges, has not yet been adequately explored. In addition, existing studies have rarely investigated preprocessing-based defense strategies or considered defenses under multiple perturbation levels. Therefore, we propose a momentum-based adversarial attack method tailored for wind power forecasting and introduce a novel multi-layer iterative denoising autoencoder (MLI-DAE) as a defense mechanism. The MI-FGSM attack incorporates momentum into gradient information to generate adversarial samples, which ensures the stable convergence of original clean samples into stealthy and destructive adversarial samples. The MLI-DAE operates on the principle of iterative training. It first generates adversarial samples using multiple perturbation strengths. These are then combined with clean samples and sequentially fed into a denoising autoencoder (DAE) for iterative refinement. When strategically placed as a preprocessing module before the wind power forecasting model, the trained MLI-DAE effectively mitigates adversarial attacks across varying perturbation strengths. The framework of the entire process is depicted in Figure 1. When the wind power forecasting system is subjected to adversarial attacks, the generated adversarial input samples may mislead the forecasting model into producing inaccurate forecasts, potentially causing the control center to issue wrong instructions. However, by leveraging the preprocessing capabilities of the MLI-DAE, these adversarial input samples can be reconstructed into their clean forms. This step enables the control center to maintain correct decision-making and ensure operational integrity.

The primary contributions of this paper are outlined as follows:

1. We propose an MI-FGSM attack algorithm for wind power forecasting. Compared to FGSM, MI-FGSM produces smaller perturbations to input samples while causing more substantial degradation in forecast accuracy in both white-box and black-box environments.
2. We propose an MLI-DAE defense algorithm against adversarial attacks in wind power forecasting. Compared to the advanced AT, MLI-DAE more effectively mitigates forecast errors caused by adversarial attacks with multi-level perturbation strengths, while better maintaining the original accuracy of the forecasting model.
3. The adversarial defense performance is systematically evaluated under both white-box and black-box attack scenarios in wind power forecasting. The defense model trained in the white-box environment maintains strong effectiveness against black-box attacks with different perturbation strengths, highlighting its generalization capability across diverse attack settings.

The remainder of the paper is structured as follows: Section 2 presents a comprehensive description of the DL-based wind power forecasting model. In Section 3, we analyze the attack environments and objectives and provide a detailed description of the proposed attack algorithm. Section 4 provides an overview of the proposed defense algorithm. In Section 5, we validate the effectiveness of the proposed attack and defense algorithms through a series of experiments, with a detailed comparison to existing methods. Section 6 summarizes the main conclusions of this study.

## 2. DL-Based Wind Power Forecasting

### 2.1. Forecasting Task

DL-based wind power forecasting models typically leverage historical wind power data and relevant external factors to predict future wind power output. The complete historical dataset is generally divided into a training set and a testing set. The training set is denoted as $D_{tr}={\left\{\left(x_{t-h},\ldots ,x_{t-1}\right);P_{t+k}\right\}}_{t=h}^{T_{tr}}$, where $x_{t-i}$ (1≤i≤h, where *h* stands for the length of historical data) denotes the input samples. Each input sample $x_{t-i}$ includes wind speed ($x_{t-i}^{ws}$), wind direction ($x_{t-i}^{wd}$), ambient temperature ($x_{t-i}^{Et}$), and historical wind power data ($P_{t-i}$), etc. $P_{t+k}$ represents the wind power output values corresponding to the input samples. The test set $D_{t}={\left\{\left(x_{t-h},\ldots ,x_{t-1}\right);P_{t+k}\right\}}_{t=T_{tr}+h+1}^{T_{t}}$ consists of similar sample sets, which are used to assess the forecasting performance of the models.

During the training process, we denote the forecasting model as $f_{\theta }$, which learns the mapping from the past time instances $X_{t}=\left(x_{t-h},\ldots ,x_{t-1}\right)$ to the future wind power value $P_{t+k}$. The loss function during training is defined as follows:

(1) $$\begin{array}{c} L\left(X_{t}\right)=\min_{\theta }\frac{1}{T_{tr}-h+1}\sum_{t=h}^{T_{tr}}|f_{\theta }\left(X_{t}\right)-P_{t+k}| \end{array}$$

where $T_{tr}$ represents the number of training samples, and θ represents the model parameter set.

Through repeated training, the θ can be optimized to minimize the loss function $L(X_{t})$, thus improving forecast accuracy. The optimization process is as follows:

(2) $$\begin{array}{c} \theta_{n}=\theta_{n-1}-\eta \cdot \nabla_{\theta }L\left(X_{t}\right) \end{array}$$

where η denotes the learning rate and $\nabla_{\theta }L\left(X_{t}\right)$ represents the gradient of the loss function.

### 2.2. Forecasting Model

Accurate short-term wind power forecasting is essential for the stable and efficient operation of power systems. With the continuous advancement of DL technology, these algorithms have shown remarkable performance in exploring the nonlinear relationships between external factors and wind power outputs in depth [15]. Among them, LSTM stands out for its ability to effectively capture long-term dependencies within sequential data through its gating mechanisms, making it highly effective for processing time series data [16]. This type of model has been widely employed in wind power forecasting and has demonstrated excellent performance (e.g., [13,16,33]). Additionally, existing studies [25,26] have adopted LSTM to investigate adversarial security in wind power forecasting. Therefore, this work employs this mature and well-validated model to evaluate the performance of the proposed attack and defense strategies. As detailed in Table 1, our specific forecasting model structure comprises an LSTM layer followed by multiple fully connected layers.

**Figure 2:**
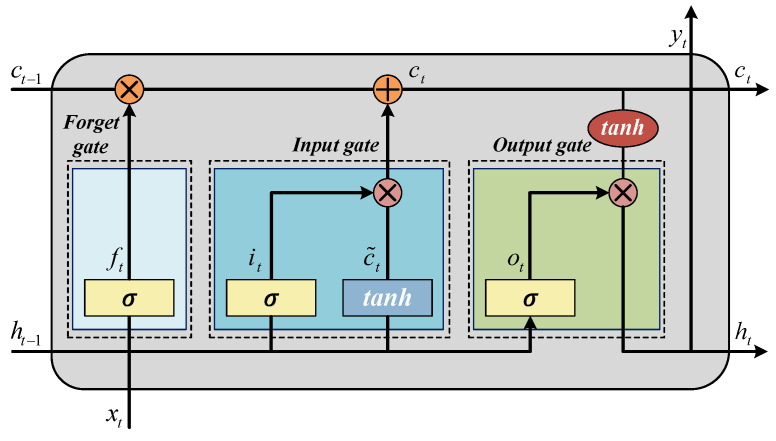
Structure of the LSTM layer.

As shown in Figure 2, the core of the LSTM layer is comprised of three distinct gating mechanisms: a forget gate, an input gate, and an output gate. With the tuning of the forget gate, the LSTM can efficiently retain and propagate information on long sequences, thus capturing long-term dependencies. Given the input data $x_{t}$, the cell state $c_{t}$ processes the time series through the following process:

(3) $$\begin{array}{c} \left\{\begin{array}{c} i_{t}=\sigma \left(W_{i}\cdot \left[h_{t-1},x_{t}\right]+b_{i}\right)\\ o_{t}=\sigma \left(W_{o}\cdot \left[h_{t-1},x_{t}\right]+b_{o}\right)\\ f_{t}=\sigma \left(W_{f}\cdot \left[h_{t-1},x_{t}\right]+b_{f}\right)\\ c_{t}=f_{t}+i_{t}\cdot \tanh\left(W_{c}\cdot \left[h_{t-1},x_{t}\right]+b_{c}\right)\\ h_{t}=o_{t}\cdot \tanh\left(c_{t}\right) \end{array}\right. \end{array}$$

where $i_{t}$ denotes the state of the input gate, $o_{t}$ denotes the state of the output gate, and $f_{t}$ denotes the state of the forget gate. $W_{i}$, $W_{o}$ and $W_{f}$ are the weight matrices of the three gates, and $b_{i}$, $b_{o}$ and $b_{f}$ are the corresponding bias vectors. σ represents the sigmoid activation function, and $h_{t}$ denotes the hidden state.

## 3. Attack Algorithms

### 3.1. Attack Environment and Objective

Adversarial attacks are generally categorized into white-box and black-box attacks [34,35], depending on the attacker’s level of knowledge and access to the target system [36]. In a white-box attack setting, the attacker has full knowledge of the forecasting model, including its architecture and parameters, and generates adversarial perturbations accordingly. This setting typically represents the worst-case attack scenario and is relevant in situations such as insider attacks, compromised cloud-based forecasting services, or leakage of deployed forecasting models. Evaluating this scenario allows us to assess the upper bound of vulnerability of wind power forecasting systems. In contrast, in a black-box attack setting, the attacker has only limited knowledge of the target model, which more closely reflects realistic attack conditions in practical applications. In this case, the attacker may train a substitute model using accessible historical wind power data and meteorological information, and exploit the transferability of adversarial perturbations to indirectly influence the target model [37]. In this black-box setting, the attacker is assumed to have no access to the target model’s internal architecture, parameters, or gradient information, and adversarial perturbations are generated solely based on the substitute model without querying gradients or internal states of the target system. To improve the attack success rate, an RNN—which shares architectural similarities with the LSTM—is employed as the substitute model. This architecture consists of an RNN layer and multiple fully connected layers. The specific structural parameters of the substitute model are detailed in Table 2. Evaluating both white-box and black-box scenarios allows us to assess model security under both worst-case and realistic threat models, thereby providing a more comprehensive security analysis framework.

The objective of the attackers is to create adversarial samples ${\hat{X}}_{t}$ by introducing adversarial perturbations in the neighborhoods of the input samples $X_{t}$ under given constraints. The goal of this manipulation is to maliciously induce the forecasting model to increase or decrease its predicted wind power output. Formally, the generation of these adversarial samples can be framed as solving the following optimization problem:

(4) $$\begin{array}{c} \begin{array}{c} L\left(X_{t}\right)=\max_{X_{t}}\gamma \cdot f\left(\hat{X_{t}}\right)\\ \mathrm{subject}\mathrm{to}\hat{X_{t}}=X_{t}+\delta_{X_{t}}\\ \;\;\;\;∥\delta_{X_{t}}{∥}_{p}\leq \epsilon \end{array} \end{array}$$

where $\delta_{X_{t}}$ represents the adversarial perturbation, and ϵ denotes the perturbation strength. The γ denotes the directional factor: when γ=−1, attackers aim to maliciously decrease the predicted wind power; when γ=1, the objective is to maliciously increase the forecasts. To ensure the generated adversarial samples remain stealthy and evade the anomaly detection system, the magnitude of the perturbation is constrained by its $L_{p}$-norm, such that ${∥\delta ∥}_{p}\leq \epsilon$, where p∈{0,1,∞} are commonly adopted norms in adversarial attacks [38,39].

In this study, the adversarial perturbation is generated over the entire input time series as a unified sequence rather than independently at each time step, allowing the temporal dependency structure learned by the forecasting model to be preserved in the perturbation pattern.

### 3.2. Fast Gradient Sign Method

FGSM is a simple but effective attack algorithm that utilizes single-step gradient information to create adversarial samples. Its principle involves introducing a single-step perturbation to the input samples along the direction of the loss function’s gradient, constrained by the $L_{\infty }$ norm, to induce obvious forecast errors. The computational procedure for this attack is formulated as follows:

(5) $$\begin{array}{c} \begin{array}{c} \delta_{X_{t}}=\epsilon \cdot sign\left(\nabla_{X_{t}}L\left({\hat{X}}_{t}\right)\right)\\ {\hat{X}}_{t}=X_{t}+\delta_{X_{t}}\\ subject\;to\;{∥\delta_{X_{t}}∥}_{\infty }\leq \epsilon \end{array} \end{array}$$

where sign(·) represents the sign function, and ${∥\cdot ∥}_{\infty }$ denotes the $L_{\infty }$ norm. The $L_{\infty }$ norm constraint ensures that each element of the input samples is perturbed independently with limited magnitude. However, due to its single-step optimization along a fixed direction, FGSM often suffers from limited attack effectiveness and insufficient stealthiness.

### 3.3. The Proposed Attack Algorithm

MI-FGSM is an iterative optimization-based attack that integrates momentum into the gradient update process. This mechanism stabilizes the update direction and mitigates the risk of converging to suboptimal local extrema. By accumulating gradients over multiple iterations, MI-FGSM identifies more effective perturbation directions, resulting in highly threatening adversarial samples [40]. Beyond its general efficacy, the momentum mechanism is particularly advantageous for wind power time series data. Unlike pixel data in image tasks, wind power time-series are continuous and exhibit strong temporal dependencies, requiring adversarial perturbations to be highly stealthy and temporally smooth. The momentum accumulation step stabilizes the gradient path, effectively preventing the perturbation from introducing abrupt, high-frequency spikes—a common artifact of non-momentum iterative methods. This stabilization ensures that MI-FGSM generates powerful yet highly concealed adversarial samples that preserve the physical smoothness of the input data, thus evading anomaly detection systems tailored for temporal irregularities.

Specifically, the algorithm first uses a momentum term *g* to accumulate gradient information from the previous n−1 iterations and the current iteration. Then, a momentum decay factor μ stabilizes the optimization direction. During each iteration, the perturbation is updated using a step size ϵ/N, and the gradient is normalized using its $L_{1}$ norm. The detailed computational procedure is as follows:

(6) $$\begin{array}{cc} & g_{n}=\mu \cdot g_{n-1}+\frac{\nabla_{X_{t}}L\left({\hat{X}}_{t,n}\right)}{∥\nabla_{X_{t}}L\left({\hat{X}}_{t,n}\right){∥}_{1}}\\ & \delta_{n}=\frac{\epsilon }{N}\cdot \mathrm{sign}\left(g_{n}\right)\\ & \mathrm{subject}\;\mathrm{to}\;∥\delta_{n}{∥}_{\infty }\leq \epsilon \\ & X_{t,n}=X_{t,n-1}+\delta_{n} \end{array}$$

where *N* denotes the number of iterations and sign(·) represents the sign function. The $L_{\infty }$ norm constraint indicates that each element of the input samples is perturbed independently with limited magnitude.

The implementation of MI-FGSM is shown in Algorithm 1. The initialization of this algorithm includes $g_{0}=0$ and ${\hat{X}}_{t,0}=X_{t}$. After *N* iterations, the algorithm can generate the adversarial sample ${\hat{X}}_{t,N}$.
**Algorithm 1:** The MI-FGSM attack against wind power forecasting     **Input**: Forecasting model: $f_{\theta }$, Testing set: $D_{t}$, Momentum decay factor: μ, Attack                 iteration number: *N*, Original input samples: $X_{t}$, Perturbation strength: ϵ,                 Step size for each attack iteration: α=ϵ/N.     **Output**: Set of the adversarial input sample: ${\hat{X}}_{T}$, Adversarial wind power                    forecasts: ${\hat{P}}_{t}$. **1**  Initialize: $g_{0}\leftarrow 0$, ${\hat{X}}_{t,0}\leftarrow X_{t}$; **2**  **for** $t=T_{tr}+h+1$ **to** $T_{t}$ **do** **3**        $X_{t}\leftarrow \left\{x_{t-h},\ldots ,x_{t-1}\right\}$; **4**        ${\tilde{P}}_{t}\leftarrow f_{\theta }\left(X_{t}\right)$; **5**    **for** 
n=0 
**to** 
*N* 
**do** **6**            Update the momentum by accumulating gradients: **7**            $g_{n}=\mu \cdot g_{n-1}+\frac{\nabla_{X_{t}}L\left({\hat{X}}_{t,n}\right)}{∥\nabla_{X_{t}}L\left({\hat{X}}_{t,n}\right){∥}_{1}}$; **8**            Calculate adversarial perturbations: **9**            $\delta_{n}=\alpha \cdot \mathrm{sign}\left(g_{n}\right)$;**10**            Introduce perturbations to input samples:**11**            ${\hat{X}}_{t,n}={\hat{X}}_{t,n-1}+\delta_{n}$;**12**      **end for**;**13**      ${\hat{X}}_{T}.\mathrm{append}\left({\hat{X}}_{t,N}\right)$;**14**  **end for**;**15**  ${\hat{P}}_{t}\leftarrow f_{\theta }\left({\hat{X}}_{T}\right)$;**16**  **return** Set of the adversarial input sample: ${\hat{X}}_{T}$, Adversarial wind power forecasts:        
${\hat{P}}_{t}$;

## 4. Defense Algorithm

### 4.1. DAE Defense Algorithm

To ensure the secure deployment of DL models, an effective strategy is to remove adversarial perturbations through preprocessing, thereby restoring the original, clean samples [31]. DAE is an enhanced form of autoencoders designed to reconstruct noise-corrupted data by employing a denoising preprocessing process. By training on noisy inputs, DAE can map adversarial samples (viewed as “noisy” inputs) back to their clean forms [41].

**Figure 3:**
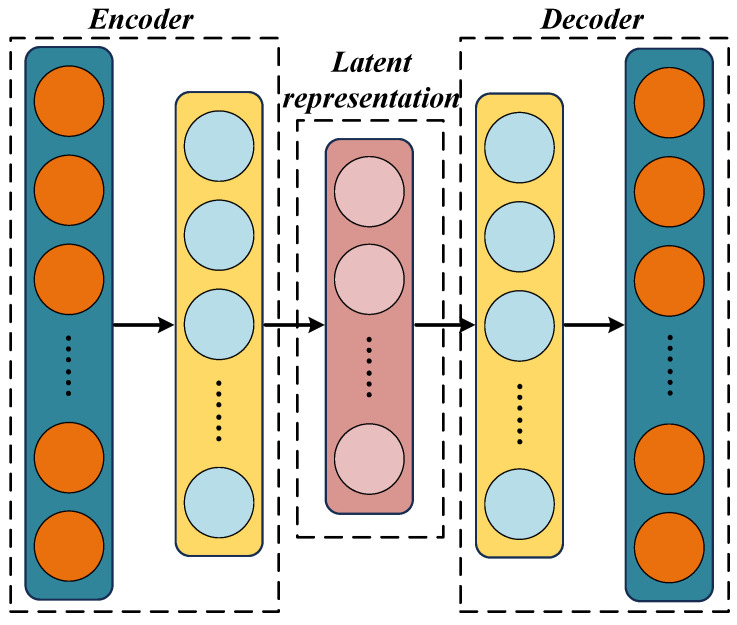
Architecture of DAE.

As illustrated in Figure 3, a DAE consists of an encoder and a decoder, both of which are constructed using fully connected layers. The encoder compresses the input samples into a latent representation, mapping from $R^{2KL}$ to $R^{z}$ through the following nonlinear transformation:

(7) $$q=\sigma (W_{1}\cdot X_{\mathrm{DAE}}+b_{1})$$

where $q\in R^{z}$ denotes the latent representation of the input sample, σ denotes the activation function, $W_{1}$ represents the weight matrix, and $b_{1}$ represents the bias vector. $X_{\mathrm{DAE}}$ represents the input of the DAE, defined as $X_{\mathrm{DAE}}\leftarrow \mathrm{concatenate}\left({\hat{X}}_{t},X_{t}\right)$. This is because the DAE should accurately reconstruct the original input samples to preserve the original accuracy of the forecasting model. Consequently, all original samples must be included in the training set.

The decoder subsequently reconstructs the data from the latent representation back to the original input space: $R^{z}\to R^{2KL}$, involving the following process:

(8) $$\tilde{X}=\sigma \left(W_{2}\cdot q+b_{2}\right)$$

where $W_{2}$ represents the weight matrix, $b_{2}$ represents the bias vector, and $\tilde{X}\in R^{2KL}$ represents the reconstructed data.

During the training phase, DAE utilizes the Adam optimization algorithm to minimize the reconstruction error. This error reflects the difference between the reconstructed samples and the original samples, and is calculated as follows:

(9) $$L\left(\tilde{X}\right)=\min_{D}\frac{1}{n}\sum_{i=0}^{n}{\left(X_{\mathrm{target},i}-{\tilde{X}}_{i}\right)}^{2}$$

where *n* denotes the size of the training dataset, and $X_{\mathrm{target},i}$ represents the expected clean output of the DAE, defined as $X_{\mathrm{target}}\leftarrow \mathrm{concatenate}\left(X_{t},X_{t}\right)$.

### 4.2. The Proposed Defense Algorithm

DAE typically exhibits excellent defense performance against single-level noise [42]. However, attackers can generate adversarial samples with diverse perturbation strengths, which may degrade the mapping accuracy learned by DAE. Therefore, we propose a defense model against multi-level perturbation attacks in wind power forecasting, called MLI-DAE. By iteratively training the DAE using adversarial samples with different perturbation strengths, MLI-DAE can adapt to attacks across multiple perturbation levels. Theoretically, this multi-level iterative training scheme significantly enhances the model’s generalization ability by progressively exposing the DAE to a wide spectrum of adversarial perturbation. A standard DAE trained on a single perturbation strength focuses on correcting a narrow range of noise, resulting in limited robustness. In contrast, the sequential training on increasingly strong adversarial samples forces the DAE to learn a more robust and stable mapping function. This ensures the model effectively captures the underlying structure of the clean data, even when perturbations are large or previously unseen, leading to significantly improved generalization and reliable reconstruction against multi-level attacks. Deployed as a preprocessing stage before the wind power forecasting model, the trained MLI-DAE effectively mitigates the impact of adversarial attacks.

The detailed training procedure for MLI-DAE is outlined in Algorithm 2. This approach involves generating adversarial samples with varying perturbation strengths, which are then sequentially used to train the DAE. Specifically, we employ a gradually increasing noise strategy to generate adversarial samples, which are utilized iteratively to refine the DAE’s training. The training set consists of $X_{\mathrm{DAE}}$ and $X_{\mathrm{target}}$, denoted as $X_{\mathrm{DAE}}\leftarrow \mathrm{concatenate}\left({\hat{X}}_{t},X_{t}\right)$ and $X_{\mathrm{target}}\leftarrow \mathrm{concatenate}\left(X_{t},X_{t}\right)$.
**Algorithm 2:** Training Process of the MLI-DAE defense    **Input**: Original input samples: $X_{t}$, Adversarial input samples: ${\hat{X}}_{t}$, Initial                perturbation level: $\epsilon_{0}$, Perturbation increment step: Δϵ, Number of                
perturbation levels: *M*, Learning rate: β, Training epochs: *K*.    
**Output**: DAE model parameter set: $D^{\left(W_{1,2},b_{1,2}\right)}$ **1**  
Initialize: $\epsilon \leftarrow \epsilon_{0}$; **2**  **for** *m=0* **to** *M* **do** **3**        (
Create adversarial samples: **4**         $\hat{x}=\mathrm{attack}\left(x,\epsilon =\epsilon_{m}\right)$; **5**         Construct training dataset: **6**         $X_{\mathrm{DAE}}\leftarrow \mathrm{concatenate}\left({\hat{X}}_{t},X_{t}\right)$; **7**         $X_{\mathrm{target}}\leftarrow \mathrm{concatenate}\left(X_{t},X_{t}\right)$; **8**         Train the DAE defense model: **9**         **for** *k=1* **to** *K* **do****10**             (
Encoding process:**11**             $q=\sigma (W_{1}\cdot X_{\mathrm{DAE}}+b_{1})$;**12**             Decoding process:**13**             $\tilde{X}=\sigma \left(W_{2}\cdot q+b_{2}\right)$;**14**             Update model parameters:**15**             $D_{k}=D_{k-1}-\beta \cdot \nabla_{D}L\left(\tilde{X}\right)$;**16**       **end****17**       
($\epsilon_{m+1}=\epsilon_{m}+\Delta \epsilon$;**18**  **end**(**19**  **return** $D^{\left(W_{1,2},b_{1,2}\right)}$;

## 5. Case Studies

### 5.1. Dataset Description and Experimental Setup

This study uses the SDWPF dataset [43], provided by Longyuan Power Group Co., Ltd., which gained prominence during the Baidu KDD Cup 2022. The dataset comprises records from 134 wind turbines over a 245-day period, incorporating features such as temperature, wind speed, and wind direction. The data are recorded at a 10-min resolution. Based on previous research, to evaluate the performance of the proposed methods, a wind turbine is randomly selected to evaluate the performance of the proposed attack and defense methods. Of the entire dataset, 80% is used as the training set, while the remaining 20% serves as the testing set.

To improve model training, the callback function is utilized to optimize both the learning rate and the number of training epochs. The learning rate is initially set to 0.01 and dynamically adjusted using a decay strategy: if the loss value does not decrease over five consecutive iterations, the learning rate is reduced to one-tenth of its current value. The number of training epochs is initially set to 80, with an early stopping strategy applied: if the loss value does not decrease for 10 consecutive iterations, training is terminated early. The Adam optimizer is used for all experiments. All model training is conducted using TensorFlow and Keras in the environment equipped with NVIDIA GeForce RTX 3050 GPUs (NVIDIA: Santa Clara, CA, USA).

### 5.2. Analysis of Key Factors in the Attack Algorithm

The MI-FGSM attack algorithm generates adversarial samples by integrating a momentum optimization mechanism into the iterative attack process, where the decay factor μ serves as a critical parameter. When μ=0, MI-FGSM loses its momentum effect and degenerates into the regular iterative attack, which may lead to overfitting of the adversarial samples and consequently degrade the attack performance. Therefore, we implemented black-box and white-box attacks on the test set to determine the optimal value of the decay factor in both environments.

**Figure 4:**
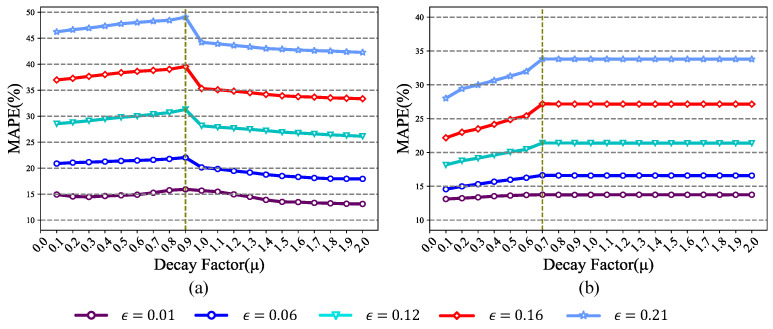
Performance of the MI-FGSM attack algorithm under different momentum decay factors: (**a**) MAPE of the forecasting model under the MI-FGSM white-box attack; (**b**) MAPE of the forecasting model under the MI-FGSM black-box attack.

The decay factor μ is incrementally varied from 0 to 2 with a step size of 0.1, the perturbation strength ϵ is set to {0.01,0.06,0.12,0.16,0.21}, and the number of attack iterations *N* is set to 60. Figure 4 illustrates the attack performance under different momentum decay factors, using the mean absolute percentage error (MAPE) as the evaluation metric. As shown in Figure 4a, the MAPE of the forecasting model under white-box attacks reaches its peak at μ=0.9 across various perturbation strengths. Figure 4b shows the MAPE of the forecasting model under the MI-FGSM black-box attacks. Under different perturbation strengths, the MAPE reaches its maximum value when μ=0.7. Therefore, we set μ=0.9 in the white-box environment and μ=0.7 in the black-box environment.

### 5.3. Experimental Analysis of MI-FGSM in White-Box Environment

**Figure 5:**
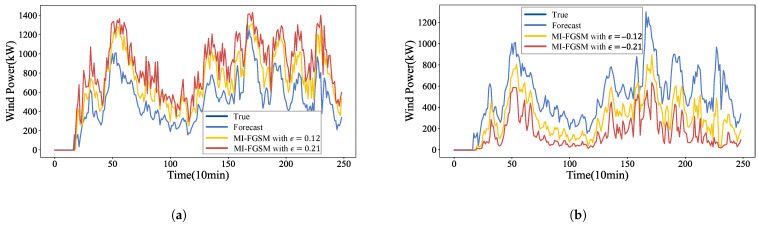
Wind power forecasting under the MI-FGSM white-box attacks: (**a**) The impact of MI-FGSM (ϵ={0.12,0.21}) on forecast curves when γ=1; (**b**) The impact of MI-FGSM (ϵ={−0.21,−0.12}) on forecast curves when γ=−1.

In the white-box environment, attackers leverage full knowledge of the forecasting model’s architecture and parameters to execute the MI-FGSM attack. To visualize the impact of MI-FGSM on wind power forecasting, we show the forecast results post-attack in Figure 5. As shown in Figure 5a, when γ=1, by injecting adversarial samples with perturbation strengths of {0.12,0.21} into the forecasting model, the forecasted values can be maliciously increased, causing the forecast curves to deviate upward from the original curve. This deviation becomes increasingly pronounced as the perturbation strength grows. Figure 5b demonstrates the scenario where γ=−1; attackers maliciously decrease the wind power forecasts by introducing perturbations with strengths of {−0.21,−0.12}. This causes the forecast curves to deviate downward from the original curve, with the deviation becoming more pronounced as the perturbation strength increases.

To demonstrate the superiority of MI-FGSM, we conduct a comparative analysis with the FGSM baseline. The experimental parameters are set as follows: (1) For FGSM, the perturbation strength ϵ is selected from {−0.2,−0.15,−0.1,−0.05,−0.01,0.01,0.05,0.1,0.15,0.2}. (2) For MI-FGSM, μ is set to 0.9, the number of iterations *N* is set to 60, and ϵ follows the same range as in FGSM. The performance of these attack algorithms is evaluated from two dimensions: attack strength and stealthiness. The attack strength assesses the extent of forecast accuracy degradation, whereas attack stealthiness quantifies the magnitude of perturbations introduced to the input samples.

To evaluate attack strength, MAPE is employed to quantify forecast errors under attacks, as shown in Table 3. The results demonstrate that MI-FGSM outperforms FGSM in terms of attack potency under the same attack directions and perturbation strengths. For example, at a perturbation strength of 0.21, MI-FGSM yields a MAPE of 49.05%, significantly higher than the 38.61% produced by FGSM. When the perturbation strength is −0.21, MI-FGSM achieves a MAPE of 40.26% compared to 32.37% for FGSM.

Stealthiness is a critical metric for evaluating the superiority of adversarial attack algorithms. Since excessive perturbations are susceptible to detection by anomaly detection systems [29,30], we quantified the perturbation percentage applied to input samples. Table 4 presents the perturbation percentages for two key input features: wind speed (WS) and wind direction (WD). The results demonstrate that MI-FGSM exhibits superior stealthiness compared to FGSM under the same perturbation strength. For instance, when ϵ=0.21, the perturbation percentages for WS and WD under FGSM are 26.91% and 29.13%, respectively; in contrast, MI-FGSM yields significantly lower values of 20.81% and 23.05%. This is because, during each iteration, the momentum mechanism of MI-FGSM guides the perturbation along the optimal attack direction, resulting in a smaller overall disturbance than that of the single-step FGSM.

Based on the analysis and comparison of experimental results in the white-box environment, it can be concluded that MI-FGSM causes the forecast curve to deviate from the original, leading to a marked decline in forecast accuracy. Compared to FGSM, MI-FGSM results in more significant forecast errors while introducing smaller perturbations to the input samples.

### 5.4. Experimental Analysis of MI-FGSM in Black-Box Environment

**Figure 6:**
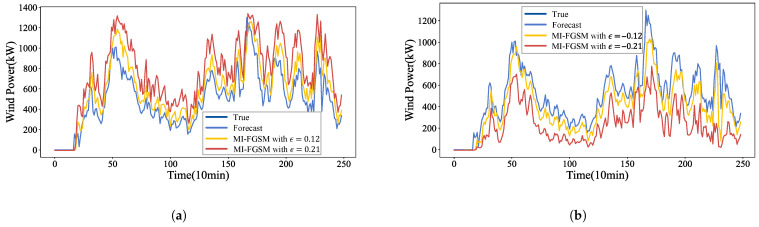
Wind power forecasting under the MI-FGSM black-box attacks: (**a**) The impact of MI-FGSM (ϵ={0.12,0.21}) on forecast curves when γ=1; (**b**) The impact of MI-FGSM (ϵ={−0.21,−0.12}) on forecast curves when γ=−1.

Black-box attacks are among the most realistic and likely scenarios in practical applications [31]. In the black-box environment, adversarial samples generated by a substitute forecasting model are employed to attack the target forecasting model. Figure 6 illustrates the forecasting curves under perturbations with strengths of {−0.21,−0.12,0.12,0.21}. As shown in Figure 6a, when γ=1, the forecast curve shifts upward relative to the original, with deviations becoming more pronounced as the perturbation strength increases. Conversely, Figure 6b demonstrates that when γ=−1, the forecast curve shifts downward, with deviations similarly amplifying alongside greater perturbation strengths. These findings indicate that MI-FGSM black-box attacks significantly deteriorate forecast accuracy, highlighting the strong transferability of the generated adversarial samples.

To facilitate a comparative analysis in the black-box environment, the parameter configurations are kept consistent with Section 5.2, with the exception of the MI-FGSM momentum decay factor μ, which is set to 0.7. Table 5 summarizes the impact of MI-FGSM and FGSM on forecasting performance. It is evident that, under the same attack direction and perturbation strength, MI-FGSM more effectively reduces forecast errors. For instance, when ϵ is 0.20, MI-FGSM results in a MAPE of 33.82%, compared to 29.12% for FGSM; similarly, at ϵ=−0.20, MI-FGSM yields a MAPE of 26.44%, whereas FGSM results in 23.63%. These findings indicate that MI-FGSM possesses superior transferability in black-box scenarios, potentially posing a severe threat to the forecasting systems. Furthermore, a comparison with Table 3 reveals that black-box attacks are notably less effective than white-box attacks under equivalent conditions. This is due to the differences in the training parameters and structure between the substitute model and the original model.

We further investigate the stealthiness of black-box attacks. Table 6 compares the perturbation percentages for WS and WD induced by MI-FGSM and FGSM. It is evident that MI-FGSM modifies the data to a lesser extent, demonstrating superior stealthiness. For instance, when ϵ=0.21, the perturbation percentages for WS and WD under FGSM are 33.81% and 36.48%, respectively; in contrast, MI-FGSM yields significantly lower values of 25.11% and 27.95%.

Based on the analysis and comparison of experimental results in the black-box environment, it can be concluded that MI-FGSM exhibits excellent ability of adversarial sample migration, causing the forecast curves to deviate from the original curve. Compared to FGSM, MI-FGSM leads to a greater increase in forecast errors, indicating its stronger black-box transferability. Additionally, MI-FGSM generates smaller perturbations to input samples, highlighting its excellent stealthiness.

### 5.5. The Defense Performance of MLI-DAE in the White-Box Environment

The procedure of the proposed MLI-DAE is shown in Algorithm 2. To evaluate the effectiveness of the MLI-DAE defense algorithm, we designed two training schemes, named MLI-DAE-5 and MLI-DAE-8, to select the optimal defense model. For MLI-DAE-5, the specific parameters are as follows: The training epoch *K* is set to 50, the learning rate δ is 0.001, the activation function is set to linear, and the optimizer is set to SGD. The iterative training process is repeated five times. In each iteration, adversarial samples generated by the MI-FGSM white-box attack are used for training, with the perturbation strength ϵ increasing from 0.01 to 0.21 in five steps (step size of 0.04). For MLI-DAE-8, the number of iteration training is increased to 8. By extending the number of training epochs and reducing the step size, the model can learn more comprehensive perturbation features. All other procedures remain consistent with those of MLI-DAE-5. To demonstrate the advantages of MLI-DAE against multi-level perturbations, a traditional DAE is trained as a baseline using MI-FGSM white-box samples with a perturbation strength of 0.12. The evaluation focused on two aspects: MLI-DAE’s efficacy in restoring forecast accuracy under attack and its ability to preserve the original forecast performance on clean data.

Table 7 summarizes the effectiveness of various defense mechanisms in enhancing forecast accuracy. Due to space constraints and the high degree of similarity in defense performance across different attack directions, only the results for γ=1 are reported. It is evident that the proposed MLI-DAE algorithm significantly mitigates forecast errors induced by MI-FGSM white-box attacks, demonstrating robust defensive capabilities. Compared to the traditional DAE, MLI-DAE consistently exhibits superior performance across all perturbation strengths, whereas the baseline DAE struggles to withstand multi-level adversarial samples. For example, when the perturbation strength is 0.21, MLI-DAE-5 and MLI-DAE-8 reduce the MAPE from 49.05% to 18.68% and 22.34%, respectively, while the traditional DAE only achieves a reduction to 29.11%. Consequently, MLI-DAE is a more suitable defense model for wind power forecasting. Notably, MLI-DAE-5 outperforms MLI-DAE-8 in error reduction across various perturbation strengths. This performance gap may stem from an overfitting issue of the latter scheme; the increased number of iterations and the simultaneous exposure to a wider variety of perturbation strengths may reduce the model’s generalization ability, leading to the slightly lower defense efficiency observed in MLI-DAE-8.

To evaluate the impact of MLI-DAE and DAE on original forecast accuracy, the forecasting model equipped with defense algorithms is used to predict the original input samples, and the forecast errors are shown in Table 7 (row ϵ=0). Following preprocessing by MLI-DAE-5 and MLI-DAE-8, the MAPE increased by only 0.40% and 0.63%, respectively, whereas the implementation of the traditional DAE led to a more significant MAPE increase of 1.11%. These results indicate that MLI-DAE more effectively retains the original forecast accuracy in the absence of perturbations compared to DAE.

**Figure 7:**
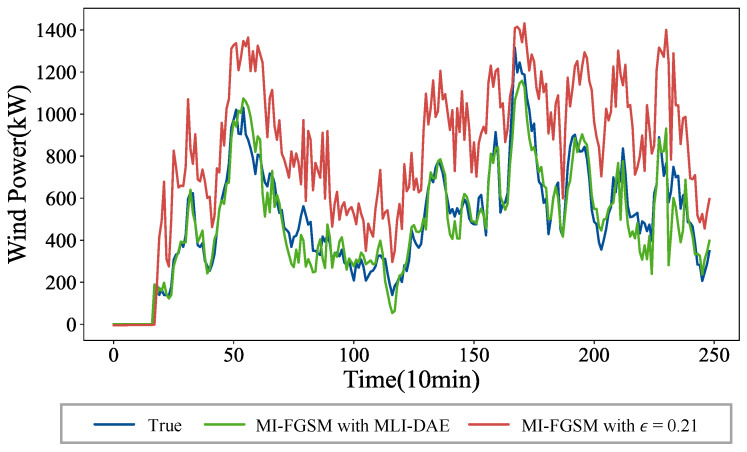
Wind power forecast results with the MLI-DAE defense against the MI-FGSM white-box attack.

To provide an intuitive visualization of the defense efficacy of MLI-DAE-5, Figure 7 shows the forecast curves under the MI-FGSM white-box attack with a perturbation strength of 0.21. As observed, the attack causes a noticeable deviation in the forecast curves from the original curve. Through the preprocessing stage of MLI-DAE-5, the wind power forecasts, which are maliciously manipulated by MI-FGSM, are effectively restored. The result demonstrates that MLI-DAE can robustly defend against MI-FGSM white-box attacks.

Based on the analysis and comparison of experimental results in the white-box environment, it can be concluded that MLI-DAE possesses exceptional defense efficacy, effectively restoring the attacked forecast curve. Compared to DAE, MLI-DAE more effectively reduces the forecast errors caused by the MI-FGSM white-box attacks, while better preserving the original forecast accuracy.

### 5.6. The Defense Performance of MLI-DAE in the Black-Box Environment

To evaluate the performance of the MLI-DAE defense algorithm in the black-box environment, we employed the MLI-DAE model trained in a white-box environment to resist black-box attacks. As illustrated in Table 8, the MLI-DAE model, trained against MI-FGSM white-box perturbations, maintains remarkable efficacy against black-box attacks generated by the same strategy. Compared to DAE, the MLI-DAE also demonstrates superior defense capabilities across various perturbation levels. For instance, when the perturbation strength is 0.21, MLI-DAE-5 and MLI-DAE-8 reduce the MAPE from 33.82% to 24.42% and 27.79%, respectively, whereas DAE only reduces the MAPE to 32.15%. Furthermore, consistent with the white-box results, MLI-DAE-5 more significantly reduces the post-attack forecast errors than MLI-DAE-8, thereby offering better defense performance. However, as observed in Table 6 and Table 8, the performance of the MLI-DAE decreases in the black-box environment compared to the white-box environment. This is because the MLI-DAE is trained in the white-box environment, while the adversarial samples generated by the MI-FGSM black-box attacks are not included in the training set, thereby reducing the performance of the MLI-DAE in the black-box environment.

**Figure 8:**
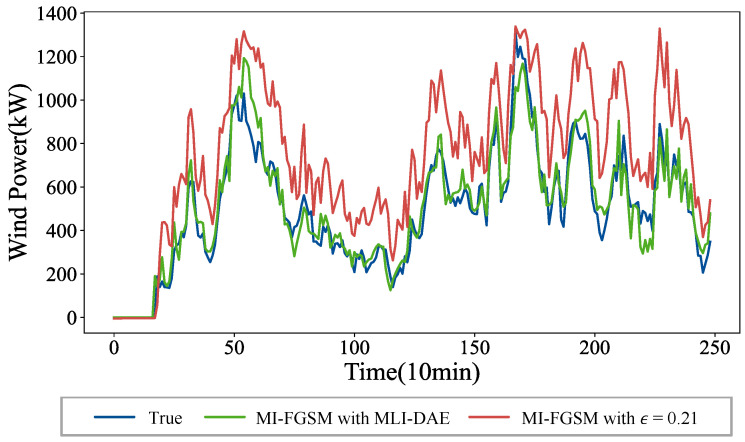
Wind power forecast results with the MLI-DAE defense against the MI-FGSM black-box attack.

To provide an intuitive visualization of the defense performance of MLI-DAE-5, Figure 8 illustrates the forecast curves under the MI-FGSM black-box attack with a perturbation strength of 0.21. The MLI-DAE-5 effectively restores the attacked forecast curves to be close to the original curve, demonstrating its effectiveness in the black-box environment.

Based on the analysis and comparison of experimental results in the black-box environment, it can be concluded that the MLI-DAE defense algorithm trained in the white-box environment can resist the MI-FGSM black-box attacks, effectively restoring the attacked forecast curve. Compared to DAE, MLI-DAE achieves a more substantial reduction in forecast errors across various perturbation magnitudes. These results highlight the excellent generalization capability of MLI-DAE in the black-box environment.

## 6. Conclusions

In this study, an MI-FGSM attack algorithm and an MLI-DAE defense algorithm are proposed for wind power forecasting. The MI-FGSM attack algorithm utilizes the momentum optimization during attack iterations to generate stealthy, high-quality adversarial samples. The MLI-DAE defense algorithm employs an iteratively trained DAE as a preprocessor, effectively mapping adversarial examples back to their clean forms. The performance of both algorithms is evaluated through extensive experiments in both white-box and black-box environments.

Experimental evaluations demonstrate that MI-FGSM causes the forecast curves to deviate upward or downward from the original curve, with the deviation becoming more pronounced as the perturbation strength increases. Compared to FGSM, MI-FGSM achieves greater degradation in forecast accuracy, induces smaller perturbations to input samples, and generates adversarial samples with stronger black-box transferability. In terms of defense, MLI-DAE effectively mitigates the impact of MI-FGSM attacks, significantly restoring the attacked forecast curves. Moreover, the MLI-DAE defense algorithm trained in the white-box environment also demonstrates strong resistance against black-box attacks, indicating its excellent generalizability in the black-box environment. Compared to DAE, MLI-DAE outperforms in reducing forecast errors caused by the MI-FGSM attacks, while hardly affecting the original forecast accuracy.

In future work, we aim to investigate the detection algorithms for adversarial attacks in wind power forecasting, with the goal of developing a comprehensive system that includes attacks, detections, and defenses. Furthermore, a detailed analysis of the computational overhead and inference latency of the MLI-DAE defense is warranted to ensure its practical applicability in real-time forecasting environments.

## Figures and Tables

**Table 1:** Structure of the forecasting model.

Model Type	Layer	Units	Activation	Optimizer
Forecasting model	LSTM	64	Tanh	Adam
	Dense	64	ReLU	
	Dense	32	ReLU	
	Dense	16	Tanh	
	Dense	1	Linear	

**Table 2:** Structure of the substitute model.

Model Type	Layer	Units	Activation	Optimizer
Substitute model	RNN	64	Tanh	Adam
	Dense	64	ReLU	
	Dense	32	ReLU	
	Dense	16	Tanh	
	Dense	1	Linear	

**Table 3:** Impact of white-box attacks on forecast results.

White-Box Attacks	Directional Factor	MAPE (%)					
		No Attack	ϵ=0.01	ϵ=0.06	ϵ=0.12	ϵ=0.16	ϵ=0.21
MI-FGSM	γ=1	12.92	15.93	22.03	31.28	39.56	49.05
	γ=−1	12.92	13.87	18.57	26.19	34.69	40.72
FGSM	γ=1	12.92	15.72	19.84	28.02	33.39	38.61
	γ=−1	12.92	13.31	15.93	22.87	29.42	33.67

**Table 4:** Perturbation percentage of input feature under white-box attack.

White-Box Attacks	Input Feature	Perturbation Percentage (%)				
		ϵ=0.01	ϵ=0.06	ϵ=0.12	ϵ=0.16	ϵ=0.21
MI-FGSM	WS	1.58	7.86	12.75	16.76	20.81
	WD	1.89	8.53	13.67	18.31	23.05
FGSM	WS	1.61	9.12	16.58	20.84	26.91
	WD	1.93	9.84	17.53	22.26	29.13

**Table 5:** Impact of black-box attacks on forecast results.

Black-Box Attacks	Directional Factor	MAPE (%)					
		No Attack	ϵ=0.01	ϵ=0.06	ϵ=0.12	ϵ=0.16	ϵ=0.21
MI-FGSM	γ=1	12.92	13.76	16.63	20.41	27.18	33.82
	γ=−1	12.92	13.28	15.32	18.27	22.87	26.44
FGSM	γ=1	12.92	13.71	15.96	19.07	24.63	29.12
	γ=−1	12.92	13.23	14.95	17.64	20.79	23.63

**Table 6:** Perturbation percentage of input feature under black-box attack.

Black-Box Attacks	Input Feature	Perturbation Percentage (%)				
		ϵ=0.01	ϵ=0.06	ϵ=0.12	ϵ=0.16	ϵ=0.21
MI-FGSM	WS	1.67	9.49	16.31	20.59	25.11
	WD	1.95	10.03	17.19	22.17	27.95
FGSM	WS	1.72	10.22	19.97	26.23	33.81
	WD	2.13	10.96	21.02	27.69	36.48

**Table 7:** Impact of defense algorithms on forecast results in the white-box environments.

Perturbation Strength	Defense Algorithms			
	No Defense	MLI-DAE-5	MLI-DAE-8	DAE
ϵ=0	12.93%	13.33%	13.56%	14.04%
ϵ=0.01	15.93%	13.95%	13.75%	14.31%
ϵ=0.06	22.03%	14.45%	14.87%	14.93%
ϵ=0.12	31.28%	15.31%	16.01%	15.23%
ϵ=0.16	39.56%	16.58%	18.23%	22.46%
ϵ=0.21	49.05%	18.68%	22.34%	29.11%

**Table 8:** Impact of defense algorithms on forecast results in the black-box environments.

Perturbation Strength	Defense Algorithms			
	No Defense	MLI-DAE-5	MLI-DAE-8	DAE
ϵ=0.01	13.76%	14.29%	14.84%	15.56%
ϵ=0.06	16.63%	15.37%	15.51%	16.14%
ϵ=0.12	20.41%	17.26%	19.52%	18.73%
ϵ=0.16	27.18%	20.77%	23.47%	25.27%
ϵ=0.21	33.82%	24.42%	27.79%	32.15%

## Data Availability

The dataset used in this study is publicly available. The complete dataset can be accessed at: https://doi.org/10.1038/s41597-024-03427-5.
